# Quality assurance device for four‐dimensional IMRT or SBRT and respiratory gating using patient‐specific intrafraction motion kernels

**DOI:** 10.1120/jacmp.v8i4.2683

**Published:** 2007-09-17

**Authors:** Benjamin E. Nelms, Eric Ehler, Henry Bragg, Wolfgang A. Tomé

**Affiliations:** ^1^ Canis Lupus LLC Sauk County Wisconsin; ^2^ Medical Sciences Center Madison Wisconsin; ^3^ Sun Nuclear Corporation Melbourne Florida; ^4^ Human Oncology and Medical Physics University of Wisconsin Madison Wisconsin U.S.A.

**Keywords:** IMRT, SBRT, quality assurance, IMRT QA, ITV, intrafraction motion, 4D

## Abstract

Emerging technologies such as four‐dimensional computed tomography (4D CT) and implanted beacons are expected to allow clinicians to accurately model intrafraction motion and to quantitatively estimate internal target volumes (ITVs) for radiation therapy involving moving targets. In the case of intensity‐modulated (IMRT) and stereotactic body radiation therapy (SBRT) delivery, clinicians must consider the interplay between the temporal nature of the modulation and the target motion within the ITV. A need exists for a 4D IMRT/SBRT quality assurance (QA) device that can incorporate and analyze customized intrafraction motion as it relates to dose delivery and respiratory gating. We built a 4D IMRT/SBRT prototype device and entered (*X*, *Y*, *Z*)(*T*) coordinates representing a motion kernel into a software application that

1. transformed the kernel into beam‐specific two‐dimensional (2D) motion “projections,”
2. previewed the motion in real time, and
3. drove a precision *X*–*Y* motorized device that had, atop it, a mounted planar IMRT QA measurement device.

The detectors that intersected the target in the beam's‐eye‐view of any single phase of the breathing cycle (a small subset of all the detectors) were defined as “target detectors” to be analyzed for dose uniformity between multiple fractions. Data regarding the use of this device to quantify dose variation fraction‐to‐fraction resulting from target motion (for several delivery modalities and with and without gating) have been recently published. A combined software and hardware solution for patient‐customized 4D IMRT/ SBRT QA is an effective tool for assessing IMRT delivery under conditions of intrafraction motion. The 4D IMRT QA device accurately reproduced the projected motion kernels for all beam's‐eye‐view motion kernels. This device has been proved to

• effectively quantify the degradation in dose uniformity resulting from a moving target within a static planning target volume, and

• integrate with a commercial respiratory gating system to ensure that the system is working effectively.

Such a device is discussed as a potential tool to optimize the gating duty cycle to maximize delivery efficiency while minimizing dose variability.

PACS numbers: 87.50.Gi, 87.53.Dq, 87.53.Kn, 87.53.Mr, 87.53.Tf, 87.56.Fc, 87.58.Sp

## I. INTRODUCTION

The prescription, treatment planning, and dose delivery of intensity‐modulated radiation therapy (IMRT) are inherently customized. The process of IMRT treatment planning includes definition of target volumes and margins, critical volumes, dose distribution, immobilization devices, and delivery parameters that are specific for each patient's anatomy and treatment goals. Until recently, the treatment planning and delivery process would always assume a “rigid body” patient anatomy. Even today's per‐fraction image‐guided radiation therapy (IGRT) practices result in daily patient repositioning to give the optimal target location relative to the treatment isocenter or isocenters.

Newer technologies, such as 4D respiratory‐gated CT or implantable, traceable markers, are allowing a new degree of customization in the realm of treatment planning and delivery: the customization of a target motion envelope or “internal target volume” (ITV). These new tools allow for new strategies to handle the clinical reality of repeatable intrafraction target motion—more specifically, respiratory motion. Of particular importance is hypofractionated stereotactic body radiation therapy (SBRT), for which fractions can be much longer but also fewer in number, making tumor coverage even more critical. One general strategy is to prescribe dose to the planning target volume (PTV) and to make best efforts to minimize interplay effects. Another strategy is to use delivery gating techniques to treat during a specific subset of the period of motion, with the duty cycle directed around the time of least target movement. These strategies—ITV‐based prescription and gated delivery—can be used in various combinations.

As this new level of patient customization enters clinical practice, an appropriate 4D dose QA device must arrive in lock‐step. The American Association of Physicists in Medicine (AAPM) Task Group 76^(1)^ published a very useful guide to practical 4D patient management, and in it, they advise an individualized approach to respiratory management, particularly suggesting that the motion pattern and treatment methods must be studied individually for each patient. Previous studies of the dosimetry to a moving target and respiratory gating systems have not incorporated patient‐specific motion kernels.^(^ ^2^ ^–^ ^4^ ^)^ A useful device would allow for

- assessment of fraction‐to‐fraction dose variation for a given ITV and intensity‐modulation technique;
- selection of the safest delivery option—step‐and‐shoot multileaf collimation (SMLC), dynamic multileaf collimation (DMLC), compensator‐based IMRT, and so on—for any given patient;
- determination of the optimal ITV;
- data‐driven decisions on whether gating is required, and the optimal duty cycle;
- patient‐specific verification and QA of delivery gating; and
- pre‐treatment dry runs to consider practical data such as delivery times, especially for hypofractionated treatment.

The ideal 4D dose QA device can be imagined. It would have a large number of very small (sub–cubic millimeter), isotropic‐responding, absolute dose detectors embedded as a three‐dimensional (3D) array in a water‐equivalent phantom. Internal subvolumes of this ideal device could be programmed to move as a function of time, perhaps even to move independently to allow for the simulation of deformation of the rigid body precept. Or, without any physical movement, a 3D array of real‐time signals could be used to reconstruct the cumulative dose to hypothetical dynamic tumorlets. Such a device could be used for any treatment modality (conventional static beam gantry angles, arc therapy, tomotherapy, radiosurgery, etc.). If the detector density was sufficient, measured dose–volume histograms (DVHs) could be estimated by treating the detectors as small voxels of tissue and dose.

However, although postulating an ideal device of the future is always important, providing evolutionary devices that can be practically used today is equally important. Technological, clinical, and practical requirements must be considered before all else when designing clinical tools. The AAPM TG‐76 report generalizes some requirements for a 4D QA device. For example:

- The ability to reproduce cyclical motion similar to human respiration
- Integration of a gating surrogate that uses the same method as clinical treatment gating
- Attachment of adequate dose detectors
- Practical reliability and cost

In the present work, we propose an actual device that meets these general requirements, plus more specific requirements. Particularly, Table 1 summarizes our more specific requirements for a 4D dose QA device that would have immediate application and impact. The requirements of Table 1 were the starting point for this work. Our goals were to build such a device and then to assess its capabilities.

**Table 1:** Some practical requirements for a four‐dimensional intensity‐modulated (IMRT) / stereotactic body radiation therapy (SBRT) quality assurance device

Requirement	Explanation
1. Electronic (filmless)	Given the resource and time constraints of modern clinics, any new device—hardware or software—should hold efficiency paramount. In intensity‐modulated quality assurance, this requirement implies filmless dosimetry to avoid processing and scanning time.
2. High resolution (sub‐mm^3^ detector size)	Intrafraction motion kernels are typically on the order of several centimeters in the direction of maximum motion, and motion cycles can be fast. To capture dosimetry accurately, volume averaging must be avoided. A small active detector size is therefore required.
3. Sufficient detector density (detectors/area)	To capture sufficient data and generate meaningful statistics, the number of detectors per unit area (two‐dimensional) must be maximized within practical device limits.
4. Quantify absolute dose	Absolute dose errors are necessary to build a clinical database and to make any assumptions about biologic effects using today's strategies.
5. Programmable motion	The detectors must move, and they must be able to move in exact or very close correlation with the beam's‐eye‐view of the target motion. Conformal radiation therapy is very customized, and a four‐dimensional dose quality assurance device must also be customizable. Motion acceleration and speed must accommodate the full range of breathing motion.
6. Integration with gating methods	Gating methodologies use different fiducial and image triggers. A programmable four‐dimensional dose quality assurance device that moves in two‐dimensions must be able to integrate with standard gatingmechanisms.
7. Integration with existing two‐dimensional array devices	To help reduce costs while still making new technologies available, new devices should, if possible, integrate with existing tools. A four‐dimensional dose quality assurance device should integrate with an existing, accepted two‐dimensional array product.
8. Use of familiar analysis methods	A four‐dimensional dose quality assurance device should, when possible, use the language and methods accepted by medical physicists—for example, percent difference, distance‐to‐agreement, gamma statistics, and so on. This approach would accelerate understanding and appropriate application of new devices.

## II. MATERIALS AND METHODS

We chose to work with an existing commercial IMRT QA device, the MapCHECK diode array (Sun Nuclear Corporation, Melbourne, FL), which has been carefully analyzed for conventional IMRT QA.^(^ ^5^ ^,^ ^6^ ^)^ This device meets the key qualifying requirements for 4D development, as defined in Table 1. First, it uses a popular commercial device, which supports requirement 7 (integration with existing 2D array devices). Second, the diode array is filmless, providing immediate dosimetric feedback (requirement 1). Third, each diode has an active detector area of 0.64 mm^2^ in the beam's‐eye‐view [BEV (requirement 2)]. The detector density is minimally acceptable, with 221 diodes evenly spaced in the central 10 cm^2^ area (requirement 3). Furthermore, the device can be calibrated to quantify absolute dose (requirement 4), and it features the common toolset for IMRT QA data analysis (requirement 8).

Other commercial devices were considered. Two‐dimensional ion chamber arrays certainly satisfy requirements 1, 3, 4, 7, and 8, but are more limited with respect to requirement 2 (detector size and resolution). With high gradients and potentially rapid motion, submillimeter resolution is desirable, and this resolution is not possible with commercial ion chamber devices. Electronic portal imaging devices (EPIDs) show much promise in IMRT QA, and these were also considered. They have excellent detector density (requirement 3) and also satisfy requirements 1 and 8, which are of great importance. However, use of EPIDs for IMRT QA is still in the very early adoption phase, and no commercial offering yet has an absolute dose calibration, thereby hindering requirements 7 and 4. Additionally, the EPID devices would have either to be programmed to move rapidly during acquisition or else to electronically map dose to moving vectors of pixels on a stationary 2D array.

The diode array therefore seemed most practical for this study. The remaining requirements—namely, the customizable, programmable motion (requirement 5) and integration with gating methods (requirement 6)—are developed in this study.

### A. Hardware

Common conventional IMRT QA methods rely on creating verification dose planes for each beam. The treatment planning system calculates a patient beam on a flat water‐equivalent phantom, and a dose cross‐section is then extracted. An analogous plane of dose is measured with the detector array or film for comparison.

This familiar 2D methodology can be extended to analyze 4D target‐motion studies, provided that the detectors that map to a target in each beam can trace out the BEV motion trajectory. In our case, a group of diodes (“target detectors”) that correspond to beamlets that intersect the radiation target needed to physically move, tracing what each beam would “see” from its reference frame during the intrafraction motion.

**Figure 1:**
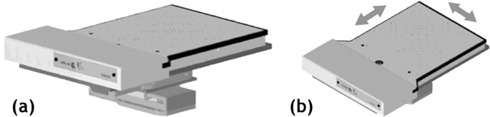
The two‐dimensional diode array mounted atop high‐precision *X*–*Y* steppers with independent motion. The *X*–*Y* steppers receive power and motion instructions from a controller device.

To achieve this movement, we used a programmable *X*–*Y* motion device (Newmark Systems, Mission Viejo, CA). The first key component was a motion controller (NSC‐M Series) to provide power and independent signals to the multi‐axis steppers. The next critical components were two independent linear stages (NLS4), each with 0.13 μm resolution, maximum travel speed of 5.08 cm/s, maximum acceleration of 72 cm/s^2^, 0.5 μm bidirectional reproducibility, and ability to carry up to a 22.7‐kg horizontal load. The devices used in this study had a 6.35‐cm travel range in *X* and *Y*. Fig. 1 is a schematic of the *X*–*Y* steppers below the diode array. A mount was built to fix the MapCHECK device securely atop the *X*–*Y* motion table. Equivalent buildup of $2{g/\text{cm}}^{2}$ and $5{g/\text{cm}}^{2}$ were tested for motion reproducibility for actual respiration motion kernels.

### B. Motion kernels

To examine realistic clinical conditions, 4D respiration‐correlated CT studies (GE HiSpeed, 4‐slice CT scanner: GE Medical Systems, Milwaukee, WI) were analyzed to quantify motion kernels for two SBRT patients. The multiphase 4D image sets were transferred into a research module of the Pinnacle^3^ treatment planning system (Philips Radiation Oncology Systems, Madison, WI). The 3D motion kernels were designed using a previously published method,^(7)^ and each ITV was created using the derived organ motion kernels.^(7)^ To account for setup error and other possible residual errors during therapy, each PTV was generated by expanding the respective ITV by a margin of 5 mm.^(8)^

### C. Programmable motion

**Figure 2:**
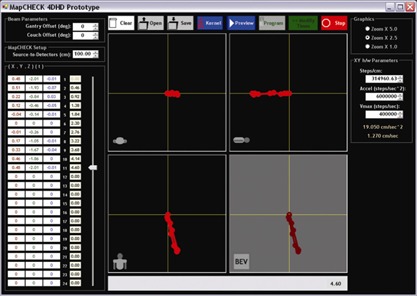
The prototype software interface, showing a three‐dimensional (*X*,*Y*,*Z*) versus time (*T*) motion kernel entry, together with axial, sagittal, and coronal projections at the plane of isocenter. Beam‐specific projections to the detector plane are also calculated and displayed.

A software application (Fig. 2 shows the user interface) was written to enter customized motion kernels and drive the motion hardware. For a given patient plan, the 3D motion kernel was entered into the system as $\left(X_{C},Y_{C},Z_{C}\right)$ in centimeters as a function of time in seconds (relative to the couch, defined in the IEC61217 patient coordinate system). Then, for each beam, the corresponding gantry and couch angle would allow the 3D motion kernel to be projected to the detector plane for any beam's specific BEV geometry. The collimator angle was not used in the transformation, because any collimator rotation could be executed for the actual QA measurements.

The conversions to BEV coordinates for any beam, considering gantry angle, couch angle, and source‐to‐detector distance are outlined next. Variables $\left(X_{B},Y_{B},Z_{B}\right)$ represent coordinates transformed to a beam coordinate system with the origin at the isocenter; γ is the gantry angle, with nominal angle of zero (vertical down beam), increasing as the gantry rotates clockwise; θ is a couch angle, with nominal angle of zero (into gantry), increasing as the couch rotates clockwise (viewed from above); and $\left(X_{\text{BEV},\text{detector plane}},Y_{\text{BEV},\text{detector plane}}\right)$ is the actual BEV projection to the detector plane [source‐to‐detector distance (SDD)].

(1) $$X_{B}=(X_{C}\cos\theta +Y_{C}\sin\theta )\cos\gamma -Z_{C}\sin\theta$$

(2) $$Y_{B}=Y_{C}\cos\theta -X_{C}\sin\theta$$

(3) $$Z_{B}=(X_{C}\cos\theta +Y_{C}\sin\theta )\sin\gamma +Z_{C}\cos\gamma$$

(4) $$X_{\text{BEV},\text{detector}\;\text{plane}}=X_{B}[\text{SDD}/(\text{SDD}-Z_{B})]$$

(5) $$Y_{\text{BEV},\text{detector}\;\text{plane}}=Y_{B}[\text{SDD}/(\text{SDD}-Z_{B})]$$

Based on the projection of the motion kernel for a single beam, the software application calculated and sent a set of ASCII instructions to the controller. These instructions defined vectors of motion connecting the points of the projected motion kernel with their appropriate *AT* time intervals from the respiration‐correlated CT study. The controller was operated in “vector‐motion mode” to ensure that the destination points and destination times were achieved with maximum accuracy without the need to program the complicated velocity and acceleration functions. Vector‐motion mode was an option afforded by the selected prototype hardware and software. Doses to the target detectors were tracked during dose delivery by the diode array acquisition software.

It should be noted that the software was programmed to alert the user if the acceleration or speed limits of the controller and steppers were to be exceeded. The “vector‐motion mode” previously described ensures that the (*X,Y,T*) points are reached accurately when possible within hardware constraints, and therefore the constraints must be examined prospectively and with brute force calculation. This examination was achieved by first estimating the 2D acceleration and velocity vectors that would connect the various phases of the cycle for the first (and then all subsequent) loops.

The first cycle starts from a stop; subsequent loops return to the starting point with non‐zero velocity vectors that become nearly identical for all cycles beyond the first. The velocity necessary to drive from any point $(X_{i},Y_{i},T_{i}$) to $\left(X_{i+1},Y_{i+1},T_{i+1}\right)$ was estimated using the relationship of equation 4, which was derived based on the relationships specified in equations 1 through 3. Estimation of velocity required recursive calculations, because those calculations depended on the initial velocity at each beginning (*X,Y,T*) data point. If any of the estimated velocity values exceeded the hardware maximum speed limit, an alert was issued, and the user given the option to “repair” the kernel by changing the *T* values to achieve allowable limits. No motion kernels in this study required repair.

In the equations that follow, $T_{i}$ is the time in seconds at the programmed trajectory point $(X_{i},Y_{i},T_{i});V_{i}$ is the uniform velocity in centimeters per second that is achieved between points *i* and i+1;A is the fixed acceleration limit in centimeters per second squared; $P_{i}$ is the linear position in one of the independent stepper directions (either *X* or *Y*, in centimeters) at the programmed trajectory point $i;P_{iA}$ is the position between points *i* and i+1 where the velocity becomes constant and the acceleration stops (centimeters); and $\Delta T_{iA}$ is the time needed to accelerate from $V_{i-1}$ to $V_{i}$ in reaching position $P_{iA}$ (seconds).

(6) $$P_{iA}=P_{i}+(V_{i-1}•\Delta T_{iA}+0.5•A•\Delta T_{iA}^{2})$$

(7) $$\Delta T_{iA}=(V_{i}-V_{i-1})/A$$

(8) $$V_{i}=(P_{i+1}-P_{iA})/[T_{i+1}-(T_{i}+\Delta T_{iA})]$$

Substituting equations 6 and 7 into equation 8, and solving for $V_{i}$ using the quadratic formula, yields

(9) $$\begin{array}{l} V_{i}=A([T_{i+1}-T_{i}+(V_{i-1}/A)]-\{{\left[T_{i+1}-T_{i}+(V_{i-1}/A)\right]}^{2}-2/A•\\ \;{\left[P_{i+1}-P_{i}+(V_{i-1}^{2}/2A)]\right\}}^{1/2}), \end{array}$$

which is used recursively to analyze maximum velocity violations for both the *X* and the *Y* steppers.

### D. IMRT planning

To show proof‐of‐concept of the device, two different SBRT plans were created for one lung tumor and motion kernel. Each plan had the same prescription, but they used different methods of delivery:

- Open field
- DMLC (sliding window)

The purpose of this experiment was to see if the 4D IMRT/SBRT QA device could detect interfraction variability in the irradiation of a moving target, and if the variability differed between the two delivery methods.

The SBRT plans were generated with the Pinnacle^3^ treatment planning system. Each plan had 9 beams of 6‐MV photons delivering a total dose of 60 Gy (12 Gy per fraction) to the PTV in 5 fractions. Pinnacle^3^ generated the DMLC sequences for the multileaf collimator (MLC) plan.

### E. Integration with respiratory gating

The device was integrated with the Real‐time Position Manager (RPM: Varian Medical Systems, Las Vegas, NV) respiratory gating system. This system uses a video camera mounted in the treatment room to automatically detect motion of two markers on the external marker block. In a clinical situation, the marker block is placed on the patient using marks obtained during the 4D CT acquisition, and it moves up and down corresponding with inhalation and exhalation respectively. The treatment duty cycle is defined as a subset of the full up‐down motion, usually around the point of maximum exhalation.

**Figure 3:**
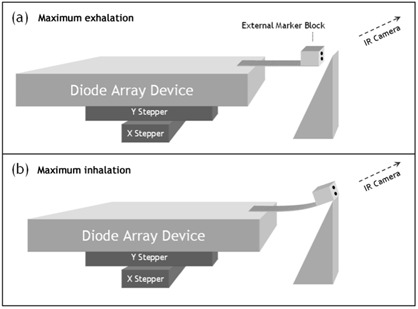
Schematic of integration with the gating system: linking the superior–inferior movement of the projected motion kernel with the gating mechanism that detects anterior–posterior movement of surrogate markers. (a) Maximum exhalation creates the most posterior marker position, and (b) maximum inhalation creates the most anterior marker position (analogous to movement of structures in the lung vs. position of markers on a breathing belt). IR=infrared.

With the 4D IMRT/SBRT QA device, inhalation and exhalation corresponded to movement along the longitudinal axis of the couch. For a patient positioned with head toward the gantry, maximum inhalation (diaphragm most inferior) was mapped to the point when the motion kernel was farthest away from the gantry. Conversely, maximum exhalation mapped to the most superior point of the motion kernel. To create the corresponding up or down motion of the marker block, an appropriate wedge apparatus was constructed (Fig. 3). As can intuitively be seen from Fig. 3, maximum exhalation (movement to the left) would create the lowest point in the up‐down marker block motion cycle.

This apparatus creates a gating trace that is not the patient breathing trace, and yet is an appropriate surrogate for the target motion. Even though this design does not yet reproduce actual patient breathing traces, the method is sufficient (per TG‐76, which states that “the surrogate breathing signal only needs to indicate the phase of the breathing motion.”^(1)^).

### F. Measurements and analysis

We analyzed 1 of the 9 beams (180‐degree planned gantry angle) from the SBRT plan so as to study the usefulness of the measurement device. First, we measured the planar QA dose for irradiation over the full breathing cycle (that is, no gating) using a Varian 600C linear accelerator with 120‐leaf MLC. To quantify the variability between identical fractions attributable to the motion of the target, each delivery was repeated 10 times, starting each time at a random point in the motion cycle. We then repeated the irradiations using the amplitude gating method on a Varian 2100iX linear accelerator, gating with a 30% duty cycle centered on maximum exhalation.

To study interfraction dose variation to the clinical target volume (CTV)–in–motion, it was necessary to determine which of the target detectors corresponded to beamlets that intersected the CTV. The treatment planning system was used to create a BEV projected to the isocenter plane for the maximum exhalation phase of the 4D CT. We overlaid that BEV on the detector array to map the diodes that would be analyzed. The SBRT target volume was relatively small, and therefore only 7 target detectors had to be analyzed.

For the analysis, the measured dose for each diode was binned in a histogram for the 10 irradiations. Because the beams were intensity‐modulated, the best way to quantify variation between the fractions was to use the coefficient of variation, which is the standard deviation of the measurements for each diode over 10 samples as a percentage of the mean value of that set. We calculated the coefficient of variation for each target detector using the formula

(10) $$CV=\frac{\sqrt{\frac{1}{N-1}\sum_{i=1}^{N}{\left(x_{i}-\bar{x}\right)}^{2}}}{\bar{x}}\times 100\%.$$

It must be noted that analysis of one beam is insufficient to evaluate an entire SBRT delivery of multiple beams of varying modulation and target motion projections. However, the variation analysis of one beam does prove that a physicist could quantify and minimize target dose variation by using such a device. For clinical plans, analysis of all beams in a plan, with their respective IMRT fields and target motion projections, would be required.

## III. RESULTS

### A. SBRT tumor motion envelopes and integration with respiratory gating

**Figure 4:**
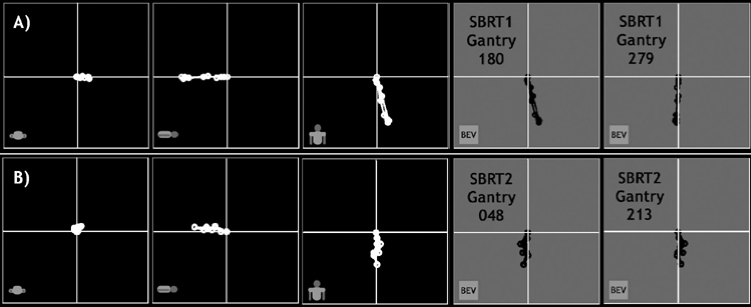
Row A): Target centroid motion kernel plots of patient SBRT1, projected to axial, sagittal, and coronal isocenter planes (panels 1 – 3). Panels 4 and 5 show two sample beam's‐eye‐view (BEV) projections for plan beams with gantry angles of 180 and 270 degrees. Row B): Target centroid motion kernel plots of patient SBRT2, projected to axial, sagittal, and coronal isocenter planes (panels 1 – 3). Panels 4 and 5 show two sample BEV projections for plan beams with gantry angles of 48 and 213 degrees.

Tables 2 and 3 show the motion kernels delineated from 4D CT studies on 2 actual patients (SBRT1 and SBRT2 respectively). Fig. 4 shows these kernels plotted in axial, sagittal, and coronal views, plus two sample “beam views” for each patient. As is typical for breathing motion, the most extreme motion is parallel to the patient superior–inferior axis.

**Table 2:** Target centroid motion kernel data for patient SBRT1 (IEC61217)

Phase	*X* (cm)	*Y* (cm)	*Z* (cm)	*T* (s)
1 (maximum exhalation)	0.00	0.00	0.00	0.00
2	−0.01	−0.26	0.00	0.46
3	0.17	−1.05	−0.01	0.92
4	0.33	−1.67	−0.04	1.38
5	0.46	−1.86	0.00	1.84
6 (maximum inhalation)	0.48	−2.01	−0.01	2.30
7	0.51	−1.93	−0.07	2.76
8	0.22	−0.84	0.03	3.22
9	0.12	−0.46	−0.05	3.68
10	−0.04	−0.14	−0.01	4.14
1 (closed kernel)	0.00	0.00	0.00	4.60

**Table 3:** Target centroid motion kernel data for patient SBRT2 (IEC61217)

Phase	*X* (cm)	*Y* (cm)	*Z* (cm)	*T* (s)
1 (maximum exhalation)	0.00	0.00	0.00	0.00
2	0.03	−0.25	0.01	0.44
3	0.03	−0.50	0.20	0.88
4	−0.10	−0.88	0.15	1.32
5 (maximum inhalation)	0.04	−1.40	0.21	1.76
6	−0.10	−1.01	0.07	2.20
7	0.13	−0.89	0.19	2.64
8	0.07	−0.63	0.08	3.08
9	0.21	−0.50	0.24	3.52
1 (closed kernel)	0.00	0.00	0.00	3.96

**Figure 5:**
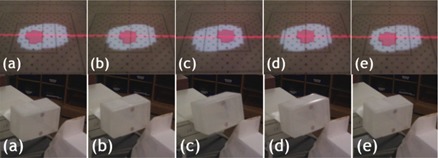
Photographs of the positions of the moving detectors inside the internal target volume (top row). The target detectors are those overlapping with the smaller clinical target volume projection. These photographs were captured at these approximate time points: (a) 0 s, (b) 0.7 s, (c) 2.3 s, (d) 3 s, and (e) 4.6 s. The bottom row shows photographs of the external gating markers and how they move in correlation with the projected motion kernels.

**Figure 6:**
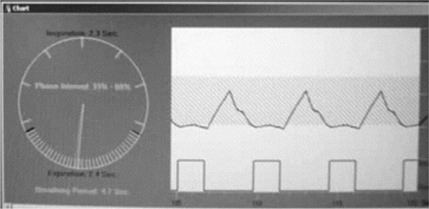
Real‐time Position Manager (RPM: Varian Medical Systems, Palo Alto, CA) respiratory gating console. The upper window shows a real‐time camera image of the two RPM markers, caught in time during their up–down cycle. The lower window plots the vertical rise and fall of the marker position versus time and the duty cycle indicating when the delivery is turned on and off. Here, the pulses are turned on when the markers are lowest, representing the phase centered on maximum exhalation.

For SBRT1, plan beam of gantry 180 degrees, the PTV (ITV plus setup uncertainty) and interior moving gross tumor volume (GTV) are shown in Fig. 5. Five time points of this particular breathing cycle are sampled from a live movie of the device cycling through the motion. The light field represents the PTV, stationary in space, while the GTV (shown as an interior projection) moves around through the projected motion kernel. Also in Fig. 5, these same five time points are shown driving the markers necessary for the RPM respiratory gating system. Finally, Fig. 6 shows a screen capture of the RPM console, where the 30% duty cycle is seen centered on the point equivalent to maximum exhalation.

### B. Reproducibility and accuracy of motion trajectories

It was important to verify that the motion was accurate and reproducible over many cycles. The first prototype of the product used a motion method that computed the necessary accelerations and velocities to move from point to point in the kernel. This initial method exhibited drifts of several millimeters over 10 minutes of constant motion. We then modified the instructions to the controller to work in “vector‐motion mode” which ensured that each (*X,Y*) point in the BEV was reached with perfect accuracy, thus avoiding any positional drifts. However, although guaranteeing spatial accuracy, vector‐motion mode can cause minor, consistent deviations in the time period of each cycle (that is, time to complete cycle versus nominal cycle period). It was important to quantify these shifts to see if they were significant. We achieved this quantification by running the motion over many cycles (10 minutes’ total time) and then having the hardware report back the exact time versus the nominal time at the origin of each motion kernel. The recorded time drifts were each less than 0.01% of the total period. Table 4 summarizes the results.

**Table 4:** Motion accuracy and reproducibility of moving the four‐dimensional intensity‐modulated/stereotactic body radiation therapy quality assurance device over projected motion kernels—10 minutes constant looping, 5.10 kg load, average over 3 trials.

Patient/plan	Gantry angle (degrees)	Time drift per cycle (%)
SBRT1	180	−0.0069
SBRT1	279	−0.0074
SBRT2	48	−0.0016
SBRT2	213	−0.0005

Similar measurements were made with a 6.8‐kg load (that is, the MapCHECK device plus buildup material) with negligible differences. For example, the time drift for the 6.8‐kg load was −0.0072% as compared with −0.0069% for the 5.1‐kg load.

### C. Measurements and analysis

The first goal of the measurements was to see if the 4D IMRT/SBRT device could quantitatively analyze the variation and degradation in target dose attributable to

- intrafraction target motion or dose blurring, and
- interfractional differences because of the random interplay of MLC motion and target motion.

Today's IMRT QA relies primarily on the technique of “dose to a plane” in water‐equivalent media, and there was practical value in preserving that method. But in the 4D case, the target detectors were able to move during delivery, because the beam would “see” the movement of the target motion kernel.

**Figure 7:**
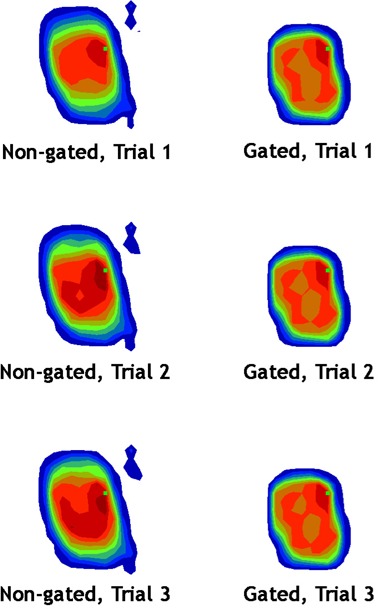
A sample of three fractions delivered to the patient SBRT1 moving target for the 180‐degree gantry angle beam. The left column (non‐gated delivery) shows not only blurring of the dose over the target diodes, but also fraction‐to‐fraction variation (that is, between the rows). The right column (gated delivery) shows that dose blurring and fraction‐to‐fraction variation are reduced.

**Figure 8:**
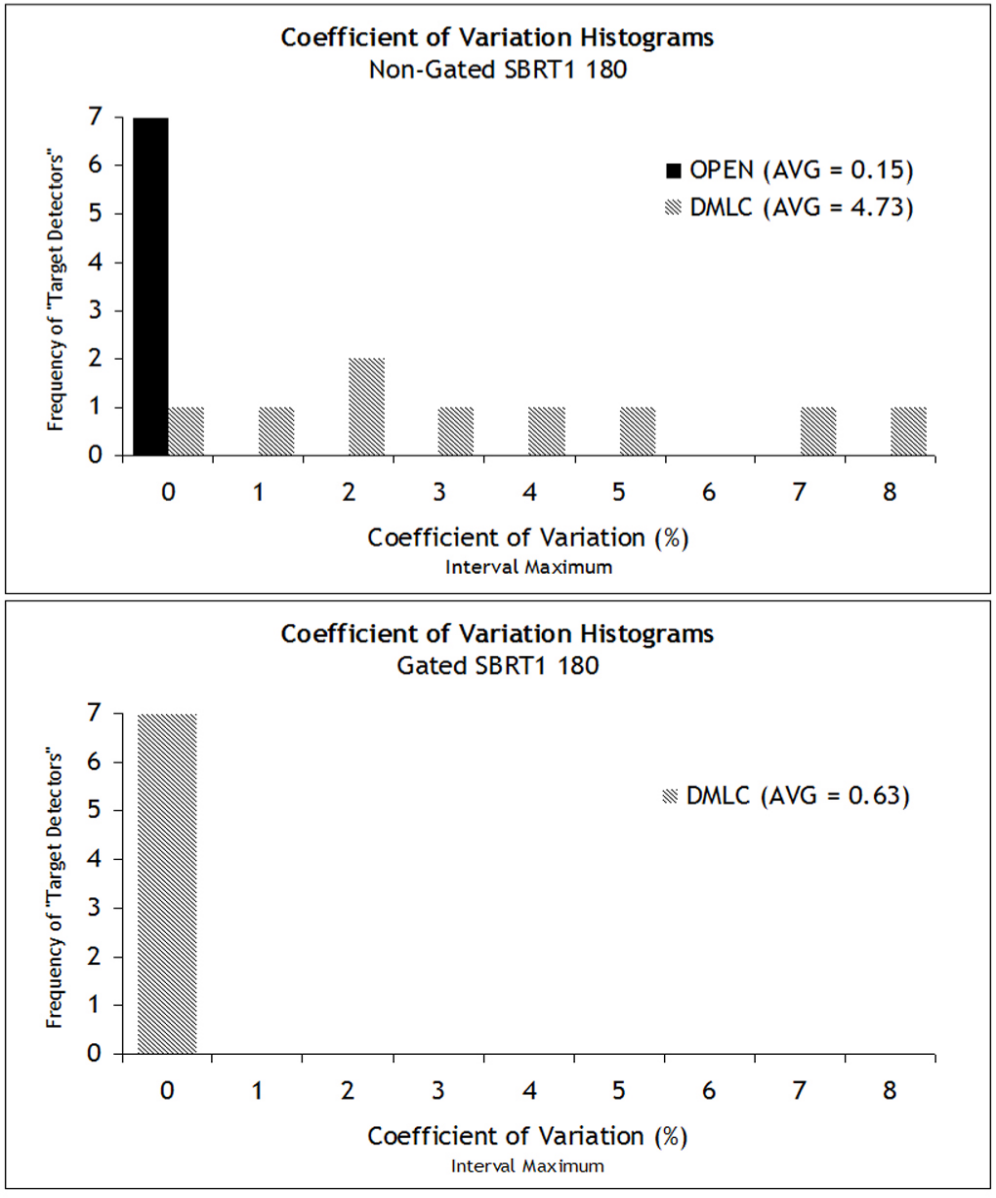
Histograms of the coefficient of variation for the target diodes of the stereotactic body radiation therapy (SBRT) 180 field measured over 10 separate fractions. Without gating, the open field delivers a much more consistent dose (that is, each target diode receives a dose that is very similar fraction‐to‐fraction) than in dynamic multileaf collimation (DMLC). When gating is employed, the coefficients of variation across the target diodes were much reduced for DMLC delivery of this beam.

Target detectors were tracked and analyzed to compare the fraction‐to‐fraction variation for non‐gated and gated delivery of DMLC‐based IMRT. Figs. 7 and 8 show the results, and it is readily apparent that this IMRT QA device can detect and quantify degradation of dose uniformity in the case of moving targets, as evidenced by the highly‐variable dose between DMLC fractions to the moving target. In addition, the device has been used in a study published elsewhere^(9)^ to specifically analyze four SBRT delivery methods:

1. 3D (non‐IMRT) fields
2. Solid compensator‐based IMRT (SIM)
3. SMLC
4. DMLC

These applications show the device and method to be very effective for quantitative analysis comparing modalities (for example, SIM versus DMLC) and gated versus non‐gated delivery.

## IV. DISCUSSION

### A. On the clinical usefulness of the device

The ability and ease with which preliminary results for clinical SBRT beams could be generated suggests that our model of a 4D IMRT/SBRT QA prototype device is effective. The device met all the criteria described in the introduction for an immediately applicable tool for the clinic that foresees planning using 4D CT image sets and dose delivery using IMRT or SBRT techniques. The device is especially useful for clinicians who utilize respiratory gating.

**Figure 9:**
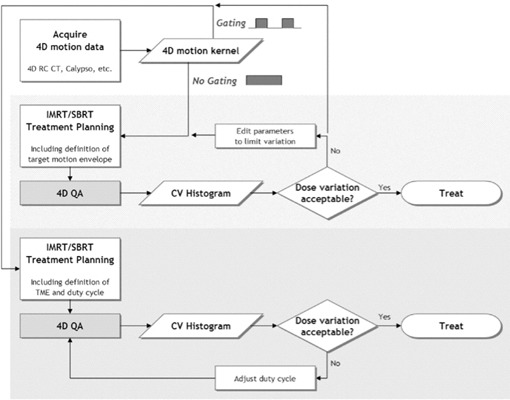
A possible decision flowchart for four‐dimensional (4D) intensity‐modulated radiation therapy (IMRT) / stereotactic body radiation therapy (SBRT) patient plans. This chart covers clinics both with and without respiratory gating technology for treatment delivery. CT= computed tomography; QA=quality assurance; CV=coefficient of variation.

However, to stringently assess the potential of such a device, an objective discussion of practical clinical usefulness is necessary. The question at issue is “What information does this device provide that will help to prove or disprove the accuracy and the safety of patient treatment?” We devote the Discussion to this important question, and we propose a potential decision flowchart for 4D IMRT/SBRT QA, as shown in Fig. 9. The figure includes decision pathways for clinics both with and without respiratory gating technology.

For clinics that do not have gating technology for treatment delivery, our device could generate data to help answer critical questions about the patient plan and delivery technique. For example, if the motion kernel data are used to define an ITV, does that ITV receive appropriate dose reproducibility, fraction‐to‐fraction, given the intrafraction motion and delivery technique? Might the target have so much variability that the goal of eradicating the clonogens is put at risk? These questions are especially critical for hypofractionated SBRT treatments, which have higher dose per fraction and few fractions. The efficacy of such treatments is far less tolerant of dose variability. By using the 4D IMRT/SBRT QA device proposed here, clinicians can prospectively quantify the intrafraction dose blurring and the interfractional variation. The clinician would therefore be empowered with data either to confidently go forward with treatment or else to stop to consider different treatment parameters, such as the delivery modality, field complexity, or breathing motion–limiting techniques.

In the case of clinics that do have gating technology for treatment delivery, this device will verify that the gating tools are working to give good dose reproducibility, fraction‐to‐fraction. Moreover, clinician will have a “dry run” to understand how long delivery of each field will take, which is very important for the scheduling of SBRT and complicated IMRT treatments for moving targets. Finally, and perhaps most interestingly, this device can serve as a tool to optimize the duty cycle of the gated deliveries—that is, it helps to answer how to achieve adequate dose reproducibility and coverage but with the most efficient duty cycle possible.

Furthermore, if a clinic with gating tools wishes to use them only when necessary, then the 4D IMRT/SBRT QA device would help in making decisions about which patients, plans, and beams require gating to achieve acceptable dose coverage. For example, a clinic could set a threshold average coefficient of variation for the target detectors. If the average coefficient of variation exceeds the threshold, gating would be employed; if the variation is under the threshold, no gating would be necessary. Each clinic would have to weigh their clinical standards and practical resources to set their requirements.

Important to the discussion of QA of gating effectiveness is the fact that this device always produces perfect correlation between the RPM markers and the detector positions. However, with actual patients, the marker and the tumor position will not be perfectly correlated.^(1)^ Our device will therefore quantify perfect gating conditions, but in reality, the clinical gains of gating will be less than are measured with this device because of the natural imperfections of tumor position and gating markers or surrogates.

### B. Other considerations

Of course, the use of the 4D QA device inherently makes two assumptions that could be debated:

1. that a target's motion kernel loops with no variation in spatial trajectory or temporal period, and
2. that quantifying variation in the target detectors is relevant to dose variation in the patient.

Regarding the first assumption, it should be remembered that the analog of the conventional 3D IMRT plan inherently assumes a rigid body patient model. Knowing full well that the rigid body precept is questionable at best with regard to patient anatomy, clinicians use CT simulation, immobilization devices, and IGRT to minimize errors, and they rely on target volume margins to account for the rest. In the same way, when we invite 4D image or motion data into the planning process, efforts must be made to minimize variation and to reproduce planning situations as best as possible. This approach particularly includes efforts at obtaining the 4D CT scans under conditions in which the motion will be cyclical and reproducible in treatment fractions. This goal is a considerable one, and we refer readers to AAPM TG‐76 for many useful suggestions and references.^(1)^ The next subsection discusses improvements that would allow for the modeling of inconsistent or drifting tumor position versus a gating surrogate. Alternatively, if treatment gating is not being used, the imaging motion conditions should be adequate to describe all the possible ranges of motion over a whole treatment—in other words, diligence is required in defining the ITV.

The second assumption (whether dose errors between a measured QA plane and a calculated QA plane have any bearing on patient dose error) must also be understood in context. In fact, this method is already the prevalent one in use for most conventional IMRT QA using film, diode arrays, and ion chamber arrays. Of course errors between measured and calculated planar doses do not directly quantify what is really wanted: specific knowledge about the resulting deviation in tumor and normal organ voxels and corresponding DVH differences. However, beam‐by‐beam IMRT QA can be an effectively stringent method if appropriate acceptance criteria are enforced. When expanding to 4D motion models, 2D BEV‐based analyses cannot take into account the potentially changing radiologic path lengths to all the tumor voxels (which are naturally arranged in a 3D morphing grid) because of patient surface changes and non‐rigid anatomic relationships. Until practical electronic, high‐resolution, morphing 3D detector arrays are available, we must continue to glean useful information from planar QA analyses.

### C. Necessary improvements

In the present study, we manually tracked and analyzed target detectors by pulling the related data out of the measurement files. For general clinical use, this approach would not be efficient and therefore is not acceptable. It is essential that the QA software analysis tools be able to import DICOM (digital imaging and communications in medicine) radiation therapy structure sets from the treatment planning system and render structure projections for each beam. Then, analysis data filters could be designed to include only the detectors that correspond to beamlets that penetrate the defined structure for the beam. Geometric beam parameters, together with the beam isocenter relative to the structure coordinates, would therefore also be required and could be derived from the DICOM radiation therapy plan data.

This first implementation uses external markers that track anterior–posterior (*Z*) motion as a unique function of superior–inferior (*Y*) motion. Therefore, it can uniquely indicate the phase of the respiratory motion cycle as described in TG‐76. However, in its current form, it cannot reproduce the actual patient trace from a surrogate marker in or on the patient anatomy. A future enhancement would be to enable the *Z*‐axis gating surrogate to be similarly motorized and programmed to match actual (or estimated or modeled) patient breathing traces. Additionally, it would be interesting to be able to program drifts and losses of correlation between the gating surrogate and the actual target (detector) motion. These variations would be possible with an independent motion device for the gating surrogate.

This study showed that the prototype was useful for standard IMRT delivery methods—that is, solid IMRT, SMLC, and DMLC. However, only one respiratory gating system (the Varian RPM system) was integrated and tested. It will be necessary to integrate other gating techniques such as the pressure sensor system^(3)^ and the thoracic tension belt offered by Philips, plus any other viable commercial solutions.

## V. CONCLUSIONS

The 4D IMRT/SBRT QA prototype device meets the proposed requirements of a practical tool, namely:

1. The data acquisition was time‐efficient (electronic, no processing).
2. The active detector size is small and therefore of high resolution.
3. Absolute dose can be measured.
4. The detectors are moveable and able to map accurately to motion kernel projections in the BEV.
5. Integration with respiratory gating is possible.
6. An existing, popular IMRT QA array is used for measurements.
7. Existing and familiar methods of quantitative analysis are used.

This device could be an integral part of a 4D IMRT/SBRT decision flowchart that incorporates target‐specific 4D motion kernels to assess dose degradation, to quantify interfractional variation resulting from interplay between delivery and target motion, to verify the respiratory gating technique, and even to determine the optimal duty cycle considering both dosimetry and delivery efficiency.

## ACKNOWLEDGMENTS

The authors thank Virgil Willcut and Scott Brame for useful critique. This work was funded in part by Sun Nuclear Corporation (Melbourne, FL) and Canis Lupus LLC (Sauk County, WI).
